# Feasibility of detecting myocardial infarction in the sheep fetus using late gadolinium enhancement CMR imaging

**DOI:** 10.1186/s12968-017-0383-1

**Published:** 2017-09-13

**Authors:** An Qi Duan, Mitchell C. Lock, Sunthara Rajan Perumal, Jack R. Darby, Jia Yin Soo, Joseph B. Selvanayagam, Christopher K. Macgowan, Mike Seed, Janna L. Morrison

**Affiliations:** 1 Institute of Medical Science, Faculty of Medicine, University of Toronto, 1 King’s College Circle, Room 2374, Toronto, ON M5S 1A8 Canada; 2 Early Origins of Adult Health Research Group, Sansom Institute for Health Research, School of Pharmacy and Medical Sciences, University of South Australia, Frome Road, Adelaide, South Australia 5000 Australia; 3 Preclinical, Imaging and Research Laboratories, South Australian Health and Medical Research Institute, 101 Blacks Road, Gilles Plains, Adelaide, South Australia 5086 Australia; 4 Cardiac Imaging Research Group, Department of Heart Health, South Australian Health & Medical Research Institute, and Flinders University, GPO Box 2100, Adelaide, South Australia 5001 Australia; 5 Peter Gilgan Centre for Research and Learning, Hospital for Sick Children, Room 08.9714, 686 Bay Street, Toronto, ON M5G 0A4 Canada; 6 Division of Cardiology, Hospital for Sick Children, 555 University Avenue, Toronto, ON M5G 1X8 Canada

**Keywords:** Cardiovascular magnetic resonance, LGE CMR, Myocardial infarction, Fetal sheep model

## Abstract

**Background:**

Late gadolinium enhancement (LGE) cardiovascular magnetic resonance (CMR) imaging has enabled the accurate assessment of myocardial infarction (MI). However, LGE CMR has not been performed successfully in the fetus, where it could be useful for animal studies of interventions to promote cardiac regeneration. We believe that LGE imaging could allow us to document the presence, extent and effect of MI *in utero* and would thereby expand our capacity for conducting fetal sheep MI research. We therefore aimed to investigate the feasibility of using LGE to detect MI in sheep fetuses.

**Methods:**

Six sheep fetuses underwent a thoracotomy and ligation of a left anterior descending (LAD) coronary artery branch; while two fetuses underwent a sham surgery. LGE CMR was performed in a subset of fetuses immediately after the surgery and three days later. Early gadolinium enhancement (EGE) CMR was also performed in a subset of fetuses on both days. Cine imaging of the heart was performed to measure ventricular function.

**Results:**

The imaging performed immediately after LAD ligation revealed no evidence of infarct on LGE (n=3). Two of four infarcted fetuses (50%) showed hypoenhancement at the infarct site on the EGE images. Three days after the ligation, LGE images revealed a clear, hyper-enhanced infarct zone in four of the five infarcted fetuses (80%). No hyper-enhanced infarct zone was seen on the one sham fetus that underwent LGE CMR. No hypoenhancement could be seen in the EGE images in either the sham (n=1) or the infarcted fetus (n=1). No regional wall motion abnormalities were apparent in two of the five infarcted fetuses.

**Conclusion:**

LGE CMR detected the MI three days after LAD ligation, but not immediately after. Using available methods, EGE imaging was less useful for detecting deficits in perfusion. Our study provides evidence for the ability of a non-invasive tool to monitor the progression of cardiac repair and damage in fetuses with MI. However, further investigation into the optimal timing of LGE and EGE scans and improvement of the sequences should be pursued with the aim of expanding our capacity to monitor cardiac regeneration after MI in fetal sheep.

**Electronic supplementary material:**

The online version of this article (10.1186/s12968-017-0383-1) contains supplementary material, which is available to authorized users.

## Background

Myocardial infarction (MI) is one of the leading causes of heart failure [1–5]. Every year, at least one million people will have an MI in the United States [3]. Despite modern reperfusion and drug therapies, five year post-MI mortality remains over 20%, mainly due to heart failure from adverse left ventricular (LV) remodelling after MI [3–5]. In the past two decades, the development of the late gadolinium enhancement cardiovascular magnetic resonance imaging (LGE CMR) technique has allowed for the accurate and reproducible non-invasive diagnosis of MI in adult humans and animals [6–10]. LGE imaging, which uses a gadolinium chelate as an exogenous contrast agent to shorten myocardial T1, has enabled the accurate diagnosis of MI, evaluation of cardiac viability, and reliable quantification of even small areas of myocardial scar [8–12]. Despite these advances in human and animal studies, to the best of our knowledge, the use of LGE CMR to assess myocardial injury in the fetus has not been investigated. Although not currently applicable to humans, the visualization of MI in the fetus in animal research could be particularly beneficial for comparing the physiological response to MI between fetal and adult hearts and determining proof of principle for mechanical and/or pharmaceutical interventions.

In contrast to the response to MI observed in human adults, the developing fetal heart is capable of cardiac regeneration after MI [13–16]. This is consistent with the well-studied pattern of cardiac repair in neonatal rodents and in zebrafish throughout life [16, 17]. The difference between species in terms of the response to injury in relation to birth is due to the timing of the maturation of cardiomyocyte development [18–21]. Thus, in humans and sheep, where cardiomyocytes undergo maturation before birth, cardiac regeneration occurs more readily in fetal life than in the postnatal situation [22]. A fetal sheep model of MI has allowed researchers to investigate the mechanisms involved in fetal myocardial regeneration. The fetal response to MI is characterized by a distinct gene expression profile, elevated myocardial proliferation, diminished inflammation, lack of fibrosis, and better restoration of cellularity and cardiac function within 30 days when compared to adults [13–15]. Developing a method of detecting and monitoring the post-MI fetal heart could therefore help researchers study the mechanisms of cardiac regeneration in fetuses post-MI by promoting or inhibiting repair. Serial CMR could aid these investigations by allowing the visualization of cardiac tissues and the tracking of changes in infarct size and cardiac function.

Therefore, in the current study, we aimed to investigate the feasibility of LGE CMR to detect MI in the sheep fetus. We hypothesized that LGE CMR would reveal enhancement in the infarcted area of the fetal heart and allow us to reliably identify MI in utero.

## Methods

### Animal ethics approvals and housing conditions

Experimental protocols for animal work were approved by the South Australian Health and Medical Research Institute (SAHMRI) Animal Ethics Committee and followed the guidelines of the Australian Code of Practice for the Care and Use of Animals for Scientific Purposes developed by the National Health and Medical Research Council. 7 Merino ewes and their 8 fetuses were used in this study, including 4 twin pregnancies and 3 singleton pregnancies. Each ewe was housed in an individual pen in an indoor housing facility that was maintained at a constant ambient temperature of 20–22 °C and a 12 h light/dark cycle with ad libitum access to food and water.

### Surgical procedure to ligate left anterior descending (LAD) coronary artery branch

At 102 days gestation (term, 150 days), ewes underwent surgery under aseptic conditions with general anesthesia induced with diazepam (0.3 mg/kg; intravenous) and ketamine (7 mg/kg; intravenous) and maintained with inhalation of isoflurane (1–2%) in oxygen. The ewes’ level of anesthesia and health status (oxygen saturation, heart rate and end-tidal carbon dioxide) were recorded every 15 min by experienced personnel. Briefly, vascular catheters (Critchley Electrical Products, Silverwater, Australia) were inserted in the maternal jugular vein, fetal carotid artery, fetal jugular vein and the amniotic cavity as previously described [23–25]. Fetuses were randomly allocated to sham (*n* = 2) or myocardial infarction (MI; *n* = 6) groups. In the cases of twin pregnancies (4 pairs, *n* = 8), either one fetus was allocated to MI (*n* = 3) and the other to sham (*n* = 1) or not included in the study (*n* = 2) or one fetus was allocated to sham (*n* = 1) and the other was not included. After thoracotomy and exposure of the heart, lignocaine (0.2 ml) was administered intravenously to all fetuses prior to ligation of the LAD coronary artery branch. A silk suture was placed around the second diagonal of the LAD coronary artery in the MI group and tied off, while observing blanching of the heart tissue. The thoracotomy incision was tightly sutured in layers (ribs, muscle and skin). The fetus was returned to the uterus and the uterus was sutured closed. Fetal catheters were exteriorized through a small incision in the ewes’ flank. The maternal abdominal muscle and skin were then sutured and all catheters were secured on the ewes’ back [23–27]. At surgery, antibiotics were administered to the ewe (153.5 mg of Procaine penicillin, 393 mg of benzathine penicillin, 500 mg of dihydrostreptomycin; Lyppards, Adelaide, Australia) and the fetus (150 mg of Procaine penicillin, 112.5 mg of benzathine penicillin, 250 mg of dihydrostreptomycin; Lyppards). When the ewe was recovered from anesthesia, it was given analgesia (20 μg/kg, Xylazil, Troy Laboratories, Australia). Antibiotics were administered intramuscularly to each ewe for 3 days after surgery and to each fetus intra-amniotically (500 mg of ampicillin; Lyppards) for 4 days after surgery.

### Fetal blood gases

Fetal carotid arterial blood gas samples (0.5 ml) were collected daily to monitor fetal health with the measurement of PaO_2_, PaCO_2_, pH, oxygen saturation, haematocrit, hemoglobin concentration and base excess temperature corrected at 39 °C with an ABL 520 analyzer (Radiometer, Copenhagen, Denmark) calibrated for sheep blood.

### Deriving heart rate from blood pressure to trigger cardiac MRI

The fetal carotid and amniotic catheters were connected to displacement transducers and a quad-bridge amplifier (ADInstruments, Castle Hill, Australia) to record fetal blood pressure and amniotic pressure. All data were sampled at a rate of 400 Hz, digitised and recorded using Chart 4 (ADInstruments). The signal was processed in LabChart 7 (ADInstruments), which acted as a cardiac trigger to the CMR [28].

### Imaging protocol

All imaging was performed on a 1.5 T Siemens Sonata scanner (Erlangen, Germany). Each fetus was scanned twice, once immediately after the surgery (Scan 1), and once 3 days later (Scan 2). The primary method under investigation was T1-weighted segmented inversion-recovery gradient echo imaging to detect the presence of an infarct, while cine steady state free precession (SSFP) imaging was used to quantify ventricular volumes in systole and diastole. We attempted early gadolinium enhancement (EGE) imaging in a more limited sample (Table 1) [29]. The total duration of each scan was approximately one hour.

**Table 1:** A list of MRI sequences performed on each fetus at Scan 1 and Scan 2

Subject #	*Group*	Scan 1		Scan 2			
		EGE *(MI: n = 4; sham: n = 0)*	LGE *(MI: n = 3; sham: n = 0)*	EGE *(MI: n = 1; sham: n = 1)*	LGE *(MI: n = 5; sham: n = 1)*	Cine (*MI: n = 5; sham: n = 2)*	3D Body Volumetry *(MI: n = 5, sham: n = 2)*
1	MI				*x*	*x*	*x*
2	MI	*x*	*x*	*x*	*x*	*x*	*x*
3	MI	*x*	*x*		*x*	*x*	*x*
4	MI	*x*	*x*		*x*	*x*	*x*
5	MI	*x*		*x (UQ)*	*x*	*x*	*x*
6	Sham			*x*	*x*	*x*	
7	Sham	*x (UQ)*				*x*	*x*
8	MI			*Died before Scan 2*			*x*
Total		4	3	2	6	7	6

*X,* sequence was performed; *UQ*, that the images have unacceptable quality and were thus excluded from their respective analysis; MI, myocardial infarction group

The gadolinium enhanced imaging employed a two-dimensional inversion recovery scheme with a segmented SSFP readout and phase-sensitive inversion recovery (PSIR) reconstruction. 1.4 mmol of contrast agent (Gd-DTPA, Gadovist, Bayer, Canada) was administered by injection into the fetal jugular vein, assuming a fetal weight of 1.4 kg (0.1 mmol/kg). EGE imaging was performed immediately after the injection of the contrast agent, while LGE imaging was initiated approximately 7 min after injection, and completed at about 10 min post injection.

We expected the fetal heart rate to range between 120 and 200 beats per minute (bpm) [26, 30, 31]. We anticipated that the heart at this gestational age would have a LV end diastolic dimension of ~1.5 cm and a wall thickness of ~3 mm, with the infarcts measuring ~1 cm in length [13]. For the EGE and LGE imaging, we therefore used an in-plane spatial resolution of 1.2 × 1.2 mm. In order to achieve adequate signal to noise ratio, we used a slice thickness of 5 mm. At these heart rates both systole and diastole were short and we therefore targeted early diastole, using an acquisition window of around 90 ms. We experimented with inversion times for EGE and LGE and settled on typical adult inversion time of 250 ms for LGE and 180 ms for EGE. Typical imaging parameters for the GE imaging were: flip angle = 25 degrees; echo time = 4.18 ms; trigger pulse = 2; echo spacing = 9.1 ms; number of signal averages = 1; field of view = 300 mm; slice thickness = 5 mm; in-plane resolution =1.2 × 1.2 mm. The acquisition window started ~120 ms after the pulse trigger and lasted ~91 ms allowing 10 lines per segment and resulting in total acquisition times of ~3 min for 10 slices, with no parallel imaging.

Cine images of the heart were obtained during Scan 2 to evaluate ventricular function. Using localizers to determine the cardiac position and axis, ~10 contiguous short-axis cine slices of the ventricles were obtained using a standard SSFP sequence (scan parameters: flip angle = 50 degrees; temporal resolution = 53.85 ms; number of segments = 15; echo time = 1.51 ms; field of view = 300 mm; phase oversampling = 20%; slice thickness = 4 mm; in-plane resolution =1.0 × 0.9 mm; number of signal averages = 5; typical acquisition time = ~10 min). Around 7 true cardiac phases were captured, and this was interpolated by the scanner into ~15 calculated phases.

A three-dimensional (3D) SSFP sequence was used to obtain images of the whole fetal body (scan parameters: flip angle = 70 degrees; echo time = 4.79 ms; field of view = 350 mm; slice thickness = 2.7 mm; in-plane resolution =2.7 × 2.7 mm; acquisition time = 1 min 34 s).

### Post-processing of CMR images

Identification of the infarcted area from the LGE and EGE images was performed qualitatively by a single experienced observer (MS) who was blinded to the subject group. Cine images of the LV and right ventricle (RV) were processed separately with commercial software (QMass 7.6, Medis Medical Imaging Systems, Leiden, Netherlands) by another experienced and blinded observer (AD). The epicardial and endocardial borders of the LV, and the endocardial border of the RV were contoured (Additional file 1). The software calculated the end systolic volume (ESV), end diastolic volume (EDV), ejection fraction (EF), stroke volume (SV) and cardiac output (CO) of each ventricle and the end diastolic mass (EDM) of the LV. These values were then indexed to the fetal weight determined at post mortem. The cine images were also assessed for the visual presence of regional wall motion abnormality. Regional wall motion abnormality was noted to be visually apparent when the motion of any region of the heart wall is abnormally reduced (hypokinesia) or absent (akinesia) in the cine images. The 3D SSFP whole fetal body images were post-processed using Mimics (Materialise, Belgium). A combination of thresholding, filling and erasing tools were used to define the interface between high-signal amniotic fluid and low-signal fetus and uterus [32, 33]. The software constructed 3D models of the fetus and provided an estimation of the fetal body volume.

### Post mortem and tissue collection

After Scan 2 was performed, ewes were humanely killed using an overdose of sodium pentobarbitone (8 g; Vibrac Australia, Peakhurst, Australia). The uterus was removed by hysterectomy, and the fetus was removed and weighed. The heart was quickly dissected and weighed. The heart was reverse perfused through the aorta with heparin sulphate (5 ml; to prevent clotting and to flush blood from the heart) and a saturated KCL solution (5 ml; to arrest the heart in diastole). Fetal hearts were photographed and epicardial infarct length and area were estimated using ImageJ (National Institutes of Health, Bethesda, Maryland, USA) by tracing a region of interest around the damaged tissue, utilizing the scale captured in each photograph. The heart was then cut into ~9 sections and the infarct was visualized using 2,3,5-triphenyltetrazolium chloride (TTC) staining. All fetuses survived to post mortem except for one MI fetus that died before Scan 2. The data from this scan was excluded from the analysis.

### Statistical analysis

All values were expressed as the mean plus or minus the standard deviation. The correlation between estimated fetal volumes and actual fetal weights obtained from post-mortem analysis were analyzed using Pearson’s correlation and linear regression analysis. Statistical analysis was performed using GraphPad (Prism 6.0, San Diego, California, USA).

## Results

### Confirmation of infarct and fetal status

**Fig. 1:**
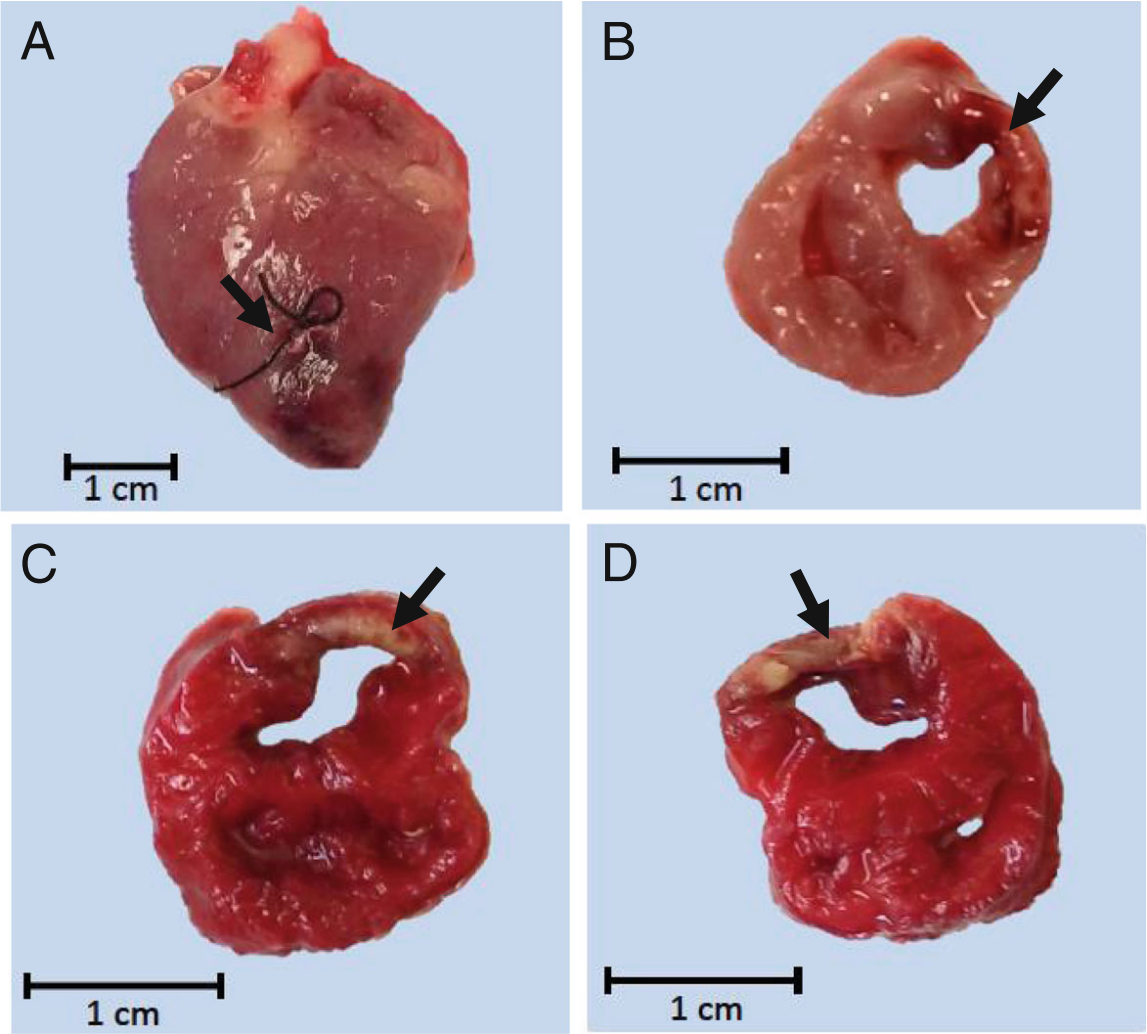
Images of an infarcted fetal heart at post mortem. **a** Infarcted fetal heart with LAD branch artery ligation at post mortem with the site of ligation visible. **b** A section of the fetal heart with infarct before TTC staining. A section of the fetal heart with infarct after TTC staining, showing **c** the basal and **d** the apical sides. Arrows indicate the infarct site

The presence of infarct in all fetuses that underwent LAD artery ligation was confirmed by TTC staining to visualise viable tissue at post mortem (Fig. 1). In five MI fetuses that survived to post mortem, the mean estimated epicardial infarct length was 1.24 ± 0.10 cm, and the mean estimated epicardial infarct area was 1.21 ± 0.19 cm^2^. The fetal blood pressure, heart rate and rate pressure product measured during the CMR session on Day 0 and Day 3 were similar in the two groups (Table 2). The blood gas status of the fetuses obtained on Day 3 was similar between the two groups (Table 3).

**Table 2:** Fetal blood pressure and heart rate during the CMR session on Day 0 and Day 3 while the ewe and fetus were anesthetised

	Day 0		Day 3	
Group	Sham *(n = 2)*	Myocardial Infarction *(n = 5)*	Sham *(n = 2)*	Myocardial Infarction *(n = 5)*
Mean Arterial Pressure (mmHg)	26.7 ± 2.3	26.4 ± 2.0	26.3 ± 5.1	27.4 ± 4.1
Heart Rate (bpm)	150 ± 19	136 ± 9	156 ± 14	170 ± 25
Rate Pressure Product (mmHg x bpm)	6052 ± 596	5261 ± 403	4580 ± 2992	6876 ± 1082

Values expressed as mean ± SD

**Table 3:** Fetal blood gas status on Day 3 in sham and myocardial infarction groups

Group	Sham *(n = 2)*	Myocardial Infarction *(n = 5)*
pH	7.37 ± 0.06	7.37 ± 0.08
P_CO2_ (mmHg)	60.1 ± 14.3	56.1 ± 13.1
P_O2_ (mmHg)	22.5 ± 0.7	18.6 ± 3.3
Hemoglobin (g/dl)	9.1 ± 0.3	7.8 ± 1.2
Oxygen Saturation (%)	45.9 ± 9.8	48.2 ± 23.7
Hematocrit (%)	28.2 ± 0.9	24.3 ± 3.6
Base Excess (mEq)	7.0 ± 1.8	5.0 ± 2.1

Values expressed as mean ± SD

### LGE imaging of the myocardial infarct

**Fig. 2:**
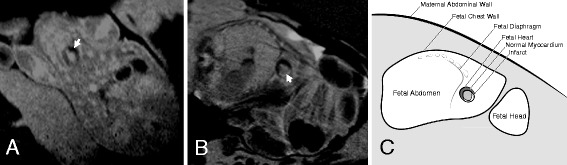
Examples of EGE and LGE images of an infarcted heart. **a** EGE images of the myocardial infarction site seen at Scan 1 and **b** LGE images of the myocardial infarction site seen at Scan 2 in the same fetus. **c** A schematic of the LGE image with maternal and fetal structures labeled. Arrows indicate the infarct site

In Scan 1, we observed no infarct on the LGE images of the three infarcted fetuses that underwent LGE CMR. However, LGE images from Scan 2 revealed clear hyper-enhancement of the infarct site in four of the five (80%) infarcted fetuses (Fig. 2b and c). These fetuses were correctly identified as having an infarct in the territory corresponding to the artery that was ligated. No sham fetus underwent LGE CMR in Scan 1. In Scan 2, the LGE images showed no infarction in one of the five infarcted fetuses; however, the quality of the LGE images of this fetus was poor. No hyper-enhanced MI area was seen in the sham fetus that underwent LGE CMR (*n* = 1) in Scan 2. This sham fetus was therefore correctly identified as having no infarct. Thus, LGE imaging is useful for detecting MI three days after the surgery, but not immediately after the surgery.

### EGE imaging of the myocardial infarct

In Scan 1, two of the four (50%) infarcted fetuses that underwent EGE imaging showed hypoenhancement at the infarct site on the EGE images. The sham fetus had non-diagnostic EGE images due to excessive artefact. In Scan 2, no hypoenhancement could be seen in the EGE images in either the sham (*n* = 1) or the infarcted fetus (*n* = 1; Fig. 2a). The EGE images of another infarcted fetus were of unacceptable image quality and the infarct was therefore not identified. These results indicate that EGE imaging is not currently reliable for detecting MI, either immediately or three days after the surgery.

### Cine imaging

**Fig. 3:**
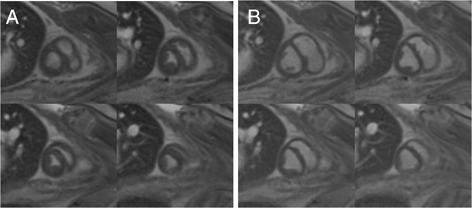
Examples of cine images of an infarcted heart. Cine images showing four slices at **a** end-systole and **b** end-diastole in an infarcted fetus at Scan 2

A regional wall motion abnormality was visually apparent in two of the five infarcted fetal hearts (Additional file 2). Indexed LV and RV function, including SV, EF, EDV, ESV, and CO, and LV EDM, of the MI group and the sham group were similar (Table 4 and Fig. 3). The average fetal heart weight measured at post mortem was 11.1 ± 2.1 g for the MI group (*n* = 5) and 11.8 ± 1.5 g for the sham group (*n* = 2).

**Table 4:** Cardiac function parameters assessed by cine imaging at Scan 2 in sham and MI fetuses

	Parameters	Sham *(n = 2)*	Myocardial Infarction *(n = 5)*
LV volumetry	LV EDM (g/kg)	2.48 ± 0.59	2.85 ± 0.39
	LV EDV (ml/kg)	3.04 ± 0.83	3.14 ± 0.33
	LV ESV (ml/kg)	1.27 ± 0.33	1.43 ± 0.46
	LV SV (ml/kg)	1.77 ± 0.50	1.71 ± 0.34
	LV EF (%)	58.2 ± 0.6	54.8 ± 11.9
	LV CO (ml/kg/min)	236 ± 4	295 ± 76
RV volumetry	RV EDV (ml/kg)	2.56 ± 0.24	3.23 ± 0.25
	RV ESV (ml/kg)	1.11 ± 0.02	1.50 ± 0.40
	RV SV(ml/kg)	1.45 ± 0.26	1.73 ± 0.37
	RV EF (%)	56.5 ± 4.9	53.6 ± 11.3
	RV CO (ml/kg/min)	198 ± 13	294 ± 69

Values are expressed as mean ± SD

*LV* left ventricular, *EDM* end diastolic mass, *EDV* end diastolic volume, *ESV* end systolic volume, *SV* stroke volume, *EF* ejection fraction, *CO* cardiac output. EDM, EDV, ESV, SV and CO are indexed to fetal weight

### CMR 3D–SSFP body Volumetry

**Fig. 4:**
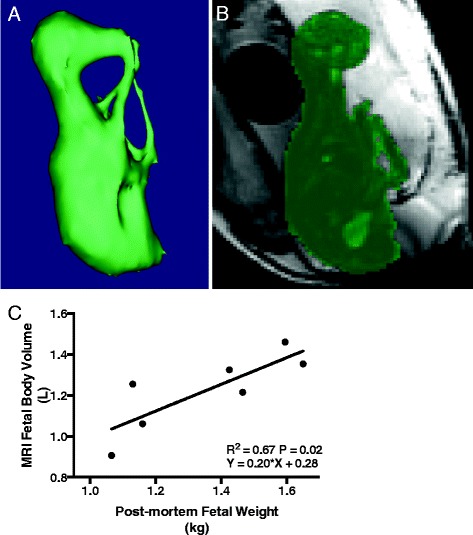
Fetal body volumetry using 3D–SSFP. Fetal body **a** segmentation and **b** volumetry of a 3D–SSFP MR acquisition. **c** A plot showing the relationship between fetal body volume measured by CMR and the fetal weight at post mortem

The average fetal weight at post mortem (immediately after Scan 2) was 1.37 ± 0.22 kg for the MI group (*n* = 5) and 1.57 ± 0.04 kg for the sham group (*n* = 2). The fetal body volumes estimated using the 3D–SSFP sequence showed a positive correlation with the actual fetal weight measured at post mortem analysis (Y = 0.20 *X + 0.28; R^2^ = 0.67; *P* = 0.02; Fig. 4). A spreadsheet including all fetal weights, epicardial infarct size, blood gas, EGE, LGE, 3D volumetry and cine results is provided in Additional file 3.

## Discussion

Sheep are one of the most commonly used large animals for research into perinatal cardiac physiology because of the similarities with humans in terms of the timing of development relative to birth, fetal size and the number of foetuses [13–15, 22]. Unlike the adult heart, the fetal heart responds to MI with a different gene expression profile, better recovery of cardiac function, diminished inflammatory response and no fibrosis [13–15]. Understanding the mechanism of fetal cardiac regeneration could help us develop ways to regenerate the myocardium in the injured adult heart. However, to date, no method has yet been developed to visualize the MI site in utero*.* It is therefore important to develop a non-invasive tool that monitors the response to MI in the fetus over time. Such a tool would allow us to visualize and potentially quantify the regenerative capacity of cardiac tissue over gestation through quantification of infarct size and cardiac function. Herein, we have shown that CMR offers a unique solution that allows us not only to monitor the infarcted area with LGE imaging, but also to assess the functional capacity of the infarcted heart with cine imaging. To the best of our knowledge, this is the first study that employs contrast enhanced imaging to detect MI in the fetus.

The implementation of CMR imaging in the fetal heart has traditionally been challenging, in part due to the difficulty in detecting the fetal heart rate for cardiac triggering. To solve this problem, several groups have developed non-invasive methods of cardiac triggering suitable for human fetuses [34–41]. Here, we employed an invasive technique similar to a method previously described by Yamamura et al. [28], and found that gating to the carotid pulse pressure was reliable. Another potential challenge to performing LGE CMR in the fetal heart arises from possible differences in gadolinium contrast handling. In normal adult cardiac tissues, the contrast agent distributes in the extracellular space, while remaining excluded from the intracellular space [42]. However, after acute irreversible myocardial injury, contrast enters the intracellular space due to disruptions in cell membranes and becomes trapped for a period of time in reperfused cardiac tissue. The fetal myocardium has different cardiomyocyte characteristics, extracellular matrix components, and response to MI, compared with the adult heart [13–15, 43]. For example, fetal cardiomyocytes are mononucleated and proliferative [44, 45]. The extracellular matrix volume is lower, and there is a reduction in wound healing and extracellular matrix deposition gene activation [15]. We were therefore uncertain about whether the gadolinium contrast agent would behave in a predictable way. However, our results show that despite known differences in microstructure and response to injury, gadolinium contrast agent is similarly trapped in the infarcted myocardium of fetal and postnatal hearts. Thus we have demonstrated, for the first time, that gadolinium contrast agent can be used in the fetus for LGE CMR.

Our results indicate that LGE CMR was successful at identifying the site of myocardial injury 3 days after ligation of a branch of the LAD coronary artery, but was unsuccessful immediately after the surgery (Day 0). This is perhaps not surprising, as LGE hyperenhancement occurs at the infarction site when the gadolinium contrast agent passively diffuses into and becomes trapped in the intracellular space after the loss of myocyte membrane integrity following necrosis [46]. We suspect that the lack of enhancement at the infarct site immediately after ligation (on Day 0) is likely because necrosis had not yet occurred at a cellular level. As a result, insufficient gadolinium had entered the intracellular space due to a lack of membrane rupture. We were therefore unable to see hyperenhancement on the LGE images. This finding is consistent with the original landmark study by Kim et al. and a later study in adult canine heart which only showed irreversible injury 4 h after artery ligation [47, 48]. On the other hand, three days after the ligation of the LAD artery, massive cell death as a result of the infarction allowed the infarcted area to trap sufficient gadolinium in the intracellular space, which eventually enabled us to see the hyper-enhanced infarct site on Scan 2.

Unlike LGE, EGE CMR is clinically useful in the setting of acute myocardial infarction, to delineate microvascular obstruction or left ventricular thrombus, which prevent the distribution of gadolinium to the infarct zone and therefore gives rise to low T1 signal [49]. Therefore, we would expect EGE CMR to show hypoenhancement in both Scan 1 and Scan 2 of the infarcted fetuses. However, in our study, EGE CMR showed microvascular obstructions in only half of the infarcted fetuses on Day 0 and none on Day 3. One possible explanation could be our compromised spatial resolution, which was necessary to achieve adequate signal to noise ratio within a reasonable scan time. Other alternative explanations are that the size of the infarcts we induced was small, whereas a microvascular obstruction is typically seen in large infarcts in humans. Finally, it is possible that we performed the second scan after resolution of microvascular occlusion, i.e. the timing of the second scan was too late to demonstrate hypo-enhancement on EGE. To fully understand the best timeline to perform LGE and EGE CMR after fetal MI, a more comprehensive study including a series of CMR scans would be needed to define the precise timeline of injury and repair, and their associated imaging characteristics.

We performed cine imaging to investigate whether regional wall motion abnormalities could be observed in the infarcted fetal hearts. We saw clear regional wall motion abnormalities in two of the five infarcted hearts. We therefore conclude that performing cine imaging is helpful in examining whether regional wall motion abnormality is present in the fetal heart. Although the infarct sizes were similar in all fetuses, we suspect the subjects with infarcted hearts that showed no regional wall motion abnormality on cine images may have had more subtle regional wall motion abnormalities that were not appreciable given the limitations of our technique.

We were also interested to see whether cine imaging could be used to quantitatively evaluate cardiac function of the infarcted fetal heart. Cine CMR is recognized as the gold standard for functional imaging and assessment of the both ventricles postnatally. However, its utility in the fetus has not been established. Yamamura et al. have previously demonstrated that cine CMR is feasible in sheep fetuses [28, 41]. They used cardiotocography as a trigger and performed cine CMR in 4 sheep fetuses (119–120 days gestation) [41]. They determined an average LVEF of 60.53 ± 4.1% and an average unindexed LVSV of 2.87 ± 0.31 ml. The LVEF and the unindexed LVSV we obtained in the sham fetuses (58.2 ± 0.4% and 2.79 ± 0.85, respectively) were similar to this prior work [41]. While we contoured the LV and RV short-axis images, we found that the most basal slice was difficult to define. This is a well-recognized problem with cine short-axis imaging [50, 51]. However, since the fetal heart was extremely small (~11g), it was particularly difficult to obtain and define the most basal slice in the short axis view with the given spatial and temporal resolution. Thus, the inclusion or exclusion of basal slices could give rise to variability in our measured ventricular volumes [50, 51]. Hawkins et al. determined a lower LVSV of 0.80 ± 0.1 ml/kg than the MRI stroke volumes using an electromagnetic flow probe at around 127 days gestation [52]. Morton et al. (137 ± 3 days) and Anderson et al. (127–133 days) also found lower LVSV using a flow probe [53, 54]. Alonso et al. used a flow probe to determine RVSV and reported a mean RVSV of 1.142 ± 0.042 ml/kg in fetal sheep of around 115 days gestation [55]. These differences in SV could be due to the variations in fetal heart size, the difference in gestational age, the lower heart rate and/or our use of anesthesia at the time of the scan, as well as variation in the accuracy of the measurement techniques. Although the mean LVSV and RVSV that we determined were higher, the heart rate we measured was lower than those reported [52–55]. Therefore, our mean fetal cardiac output (= SV x heart rate) was similar to those determined by Hawkins et al. (236 ± 4 versus 265 ± 63 ml/min/kg) and Alonso et al. (198 ± 13 versus 204.5 ± 36.8 ml/min/kg) for the left and right ventricles, respectively.

The mean EF we determined (58.2% ± 0.4%) was similar to that determined by Yamamura et al. using cine CMR and cardiotocography gating (60.53 ± 4.1%) [41]. However, these values differ from other studies that used echocardiography [13, 31]. For example, Xiong et al. used 2D echocardiography and determined a mean LVEF of 72 ± 7% in 7 control fetuses at 113 days gestation (term, 145 days), slightly higher than our results (infarction: 54.8 ± 11.9, sham: 58.2 ± 0.6) [31]. Consistent with our findings, Herdrich et al. found similar ejection fractions between fetal sheep pre-MI and post-MI (53 ± 8.1% and 54 ± 9.6%, respectively) using 2D echocardiography at 65–76 days gestation with anesthesia [13]. This variability in EF is likely due to the imaging modalities used. Measuring ventricular dimensions with 2D echocardiography is prone to error. It has been shown in previous studies that cine-MRI is superior to 2D echocardiography for determining ventricular function, demonstrating higher reproducibility and lower intra- and inter-observer variability [56–58]. Finally, an important limitation of our study was the low sample size for the sham fetuses (*n* = 2) and our results do not represent a valid set of reference ranges for normal fetal ventricular volumetry.

Major challenges to our LGE, EGE and cine imaging techniques were the fast heart rates and small size of the fetal hearts, which inevitably led to long scan times. The imaging methods we employed here are therefore not suitable for non-sedated human fetuses that move frequently. In addition, the long scan times used in this study may have also caused problems for the GE imaging relating to changes in the gadolinium concentration in the blood pool and in the myocardium during the LGE and EGE sequences. However, from a practical perspective, such variation did not appear to affect the quality of the LGE images as we were able to obtain reasonable contrast between the infarct site and the normal myocardium for each slice. While the correct inversion time for myocardial nulling was also likely to be changing during these long acquisitions, our imaging parameters resulted in good contrast between the infarct and normal myocardium. Contrast between normal and diseased myocardium was enhanced by the PSIR reconstruction used in our LGE imaging, which reduced background noise and improved the contrast-to-noise ratio [59]. The EGE sequence, however, was perhaps more affected by the long scan time, compromised spatial resolution and worse contrast-to-noise ratio, and may have contributed to its poor sensitivity. Another limitation of the study is the lack of quantification of the infarct volume in the GE images. We did not attempt to determine the infarct volume because the slices we obtained were rather thick (~ 5 mm), which was important to achieve adequate signal-to-noise ratio. As a result, the infarct was only present on one or two slices, and this precluded a reliable estimation of the volume of the infarct. The image quality was also insufficient for reliable estimation of the infarct length. However, we did estimate the epicardial infarct length seen on the heart at post mortem, and it was as we had anticipated.

We performed an analysis of our fetal body volumes obtained from the 3D–SSFP acquisitions and found excellent correlation with fetal weight at post mortem. Thus, our results support the use of fetal CMR volumetry for monitoring fetal volume over late gestation. To our knowledge, there is no established method for converting fetal volume to weight in the sheep. However, calculations of this kind have been derived for human foetuses, and a similar approach may be possible in the sheep, which would then allow fetal hemodynamic measurements to be indexed to fetal weight as they have been for invasive measurements in the past [60–62].

## Conclusion

Our results showed that LGE CMR imaging is highly feasible for the detection and monitoring of MI while EGE imaging as implemented here was less informative. Cine CMR imaging allows the visualization of regional wall motion abnormalities associated with fetal MI. However, fast fetal heart rates and the small size of the structures of interest made cine cardiac function measurements challenging and compromised their accuracy relative to adult hearts. In conclusion, our study provides evidence for the utility of a new tool for monitoring the cardiac response to MI in the fetus. However, further investigation into the optimal timing of LGE and EGE scanning and improvement of the sequences may be helpful.

## Additional files

Additional file 1: Contours of the endocardial and epicardial borders of the ventricles. A cine video of an infarcted fetal heart with the end-systolic and end-diastolic contours of the endocardial and epicardial borders of the left ventricle and the endocardial border of the right ventricle. (AVI 8445 kb)

Additional file 2: Regional wall motion abnormality of an infarcted fetal heart. A cine video showing an infarcted fetal heart with regional wall motion abnormality. (AVI 8445 kb)

Additional file 3: Dataset supporting the article. A dataset including fetal weight, epicardial infarct size, blood gas, EGE, LGE, 3D Volumetry and cine results, supporting the conclusions of this article. (XLSX 45 kb)

## Abbreviations

3D: Three-dimensional
bpm: Beats per minute
CMR: Cardiovascular magnetic resonance
CO: Cardiac output
EDM: End diastolic mass
EDV: End diastolic volume
EF: Ejection fraction
EGE: Early gadolinium enhancement
ESV: End systolic volume
LAD: Left anterior descending
LGE: Late gadolinium enhancement
LV: Left ventricle/left ventricular
MI: Myocardial infarction
PSIR: Phase-sensitive inversion recovery
RV: Right ventricle/right ventricular
SSFP: Steady state free precession
SV: Stroke volume
TTC: 3,3,5-triphenyltetrazolium chloride
